# Changes in microbial community structure and functioning with elevation are linked to local soil characteristics as well as climatic variables

**DOI:** 10.1002/ece3.9632

**Published:** 2022-12-28

**Authors:** Johannes Lux, Zhijing Xie, Xin Sun, Donghui Wu, Stefan Scheu

**Affiliations:** ^1^ J.F. Blumenbach Institute of Zoology and Anthropology University of Göttingen Göttingen Germany; ^2^ Key Laboratory of Wetland Ecology and Environment Northeast Institute of Geography and Agroecology, Chinese Academy of Sciences Changchun China; ^3^ Key Laboratory of Vegetation Ecology, Ministry of Education Northeast Normal University Changchun China; ^4^ Key Laboratory of Urban Environment and Health Institute of Urban Environment, Chinese Academy of Sciences Xiamen China; ^5^ Jilin Provincial Key Laboratory of Animal Resource Conservation and Utilization Northeast Normal University Changchun China; ^6^ Centre of Biodiversity and Sustainable Land Use University of Göttingen Göttingen Germany

**Keywords:** forest, microbes, mountain, nitrogen, organic carbon, PLFA

## Abstract

Mountain forests are important carbon stocks and biodiversity hotspots but are threatened by increased insect outbreaks and climate‐driven forest conversion. Soil microorganisms play an eminent role in nutrient cycling in forest habitats and form the basis of soil food webs. Uncovering the driving factors shaping microbial communities and functioning at mountainsides across the world is of eminent importance to better understand their dynamics at local and global scales. We investigated microbial communities and their climatic and local soil‐related drivers along an elevational gradient (800–1700 m asl) of primary forests at Changbai Mountain, China. We analyzed substrate‐induced respiration and phospholipid fatty acids (PLFA) in litter and two soil layers at seven sites. Microbial biomass (C_mic_) peaked in the litter layer and increased towards higher elevations. In the litter layer, the increase in C_mic_ and in stress indicator ratios was negatively correlated with Ca concentrations indicating increased nutritional stress in high microbial biomass communities at sites with lower Ca availability. PLFA profiles in the litter layer separated low and high elevations, but this was less pronounced in soil, suggesting that the litter layer functions as a buffer for soil microbial communities. Annual variations in temperature correlated with PLFA profiles in all three layers, while annual variations in precipitation correlated with PLFA profiles in upper soil only. Furthermore, the availability of resources, soil moisture, Ca concentrations, and pH structured the microbial communities. Pronounced changes in C_mic_ and stress indicator ratios in the litter layer between pine‐dominated (800–1100 m) and spruce‐dominated (1250–1700 m) forests indicated a shift in the structure and functioning of microbial communities between forest types along the elevational gradient. The study highlights strong changes in microbial community structure and functioning along elevational gradients, but also shows that these changes and their driving factors vary between soil layers. Besides annual variations in temperature and precipitation, carbon accumulation and nitrogen acquisition shape changes in microbial communities with elevation at Changbai Mountain.

## Graphical abstract

This paper investigated the microbial community in litter and two soil layers along an elevational gradient of primary forest at Changbai Mountain, China. We identified local soil characteristics, especially pH and calcium concentrations, as well as climatic variables as most important drivers for microbial community compositon. The influence of the elevational gradient and the relevant drivers varied between litter and soil layers.

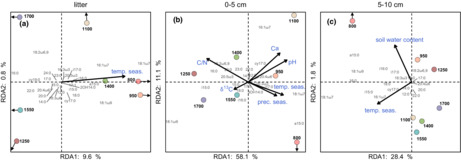

## 1 INTRODUCTION

Forests store large amounts of carbon, fixed in standing plant (tree) biomass and soil organic matter. Mountain forests contribute significantly to these carbon stocks as 41% of the worldwide mountain area is covered by forests and mountain forests sum up to 23% of the worldwide forest cover (Price et al., 2015). Global warming is expected to strongly alter mountain forests. Albrich et al. (2020) projected changes in coniferous mountain forests towards broadleaf forests at lower elevations in the European Alps. These changes in vegetation are likely to affect the structure and functioning of microbial and animal communities. Although microbial communities along elevational gradients received more attention in the last years, there is still a lack of knowledge on the factors driving microbial community composition and functioning along such gradients (Looby & Martin, 2020). Studies addressing this lack of knowledge are best to be done in mountain areas little affected by humans allowing to uncover the response of natural communities to global change factors. The northern slope of Changbai Mountain in Northeast China represents such a natural forest gradient as these forests have never been logged (Tang et al., 2011). The forests comprise mainly primary forests with a transition between deciduous and mixed forests at lower elevations towards pure coniferous and birch forests at higher elevations (Liu, 1997; Tang et al., 2011).

Litter entering the belowground system is decomposed predominantly by microorganisms, mainly bacteria and fungi (Bani et al., 2018), and therefore, microorganisms play a critical role in the mineralization of carbon and nitrogen (Hobara et al., 2014). Considering that microbial activity is intricately linked to temperature, mountains provide ideal settings to investigate the role of temperature and associated changes in forest type on the structure and functioning of microbial communities. Studying these changes is of particular relevance in face of global climate change. With decreasing temperatures and shorter vegetation periods at higher elevation, microorganisms may have to concentrate their metabolic activities to the limited period of high temperature and microbial communities have to adapt to the short period they can be active. Conform to these assumptions, Massaccesi et al. (2020) found microbial biomass to increase with increasing elevation in coniferous forests in the European Apennine indicating higher resource availability at high elevations. Changbai Mountain forests at high elevations are dominated by spruce (Liu, 1997), and spruce is known to retard decomposition processes by a high concentration of polyphenols in needles contributing to the accumulation of carbon at high elevation (Gallet & Lebreton, 1995). This is likely to be associated with distinct microbial communities.

Harsh environmental conditions including climatic and soil factors at high elevations also likely increase the physiological stress of microorganisms. Both low temperature and pH are known to result in alterations in the structure of microbial membranes (Guckert et al., 1986; Knivett & Cullen, 1965; Russel, 2008), with major consequences for microbial community composition and functioning. Conform to these considerations, Shen et al. (2013) identified pH as the main driver of changes in microbial community composition with elevation at Changbai Mountain. Effects of low temperature and pH on soil microorganisms, however, are likely to vary with soil depth due to the buffering of adverse climatic conditions by the litter layer and typically higher pH in litter than in soil. Further, the decreases in organic matter with soil depth and associated decline in resource availability (Hobley & Wilson, 2016; Kramer & Gleixner, 2008) may aggravate microbial stress in soil. Therefore, driving factors of microbial community structure and activity are likely to differ between litter and soil. As both processes in litter and soil contribute to carbon and nutrient cycling, understanding the driving factors of microbial community composition and functioning in both litter and soil is of fundamental importance. However, studies investigating changes in microbial communities along elevational gradients often only focus on soils and neglect the litter layer (Chang et al., 2016; Liu et al., 2019).

To investigate changes in microbial communities along environmental gradients phospholipid fatty acids (PLFAs) are commonly used (Chang et al., 2016; Liu et al., 2019; Xu et al., 2014). PLFAs form the major component of cell membranes and, by varying among microbial groups, provide insight into microbial community structure (Bossio & Scow, 1997; Frostegård et al., 2011; Moore‐Kucera & Dick, 2008). Further, PLFA ratios serve as indicators of environmental stress and substrate availability (Bossio & Scow, 1997; Frostegård et al., 2011; Moore‐Kucera & Dick, 2008). Thereby, PLFAs provide insight into changes in the structure and functioning of microbial communities along altitudinal gradients (Klimek et al., 2020; Liu et al., 2019). Similarly, microbial basal respiration and substrate‐induced respiration (SIR) provide insight into gross characteristics of microbial communities such as microbial biomass and activity as well as the efficiency in the use of carbon resources by microorganisms (Anderson & Domsch, 1978, 1993; Scheu, 1992).

We used PLFAs and SIR to follow changes in microbial community structure and functioning in litter and soil of forests along an altitudinal transect of Changbai Mountain, China. We hypothesized (i) microbial biomass and metabolic quotient to increase with increasing elevation but to decrease with soil depth; (ii) microbial community composition, represented by PLFA profiles, to change with elevation and soil depth, with the changes being less pronounced in soil than in litter; (iii) elevation‐related climatic variables and pH to be the major factors structuring microbial communities in litter, while in soil local soil characteristics to be most important; and (iv) physiological and nutritional stress indicators to increase with increasing elevation (due to increased environmental harshness) and soil depth (due to increased resource shortage).

## 2 MATERIAL AND METHODS

### 2.1 Study site and sampling

**FIGURE A1:**
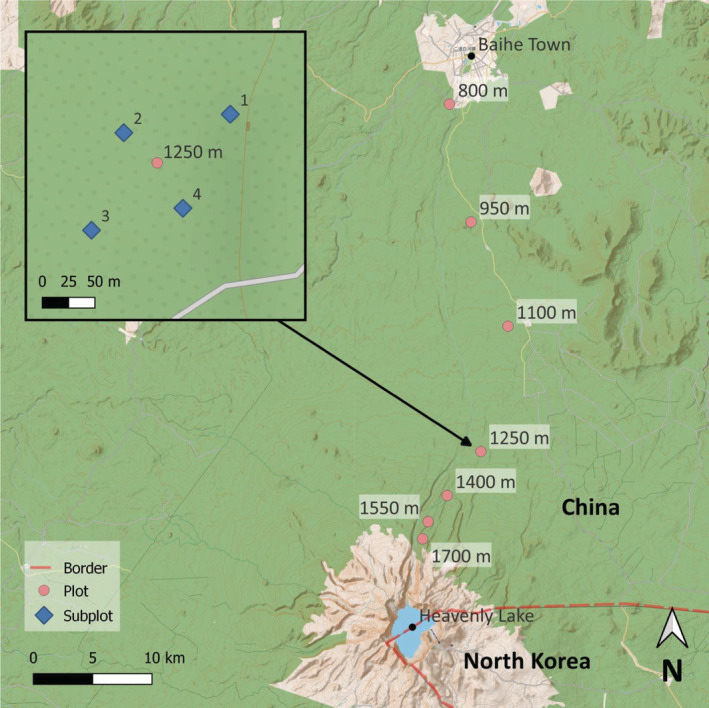
Map of the sampled transect at the northern slope of Changbai Mountain. The enlarged section displays the subplot structure for one of the elevations. Map data from “OpenTopoMap” (Erhardt et al., 2022).

Changbai Mountain (42°8'25.4004”N, 128°7'36.2352″E) extends along the border between the Chinese provinces Jilin and Liaoning and North Korea, with the “Changbaishan” being the highest mountain (2750 m asl). Samples were taken along the northern slope of the mountain forming part of the “Changbaishan National Nature Reserve.” The alkaline geological groups in the sampling area comprise stomatal and laminated basalt, alkali pumice, trachyte and tuff, reflecting the volcanic history of the mountain (Yan et al., 2018). The area belongs to the temperate climate regime and is characterized by long winters and short and warm summers. Between 1959 and 1988, the annual mean temperature ranged from −7 to 3°C and precipitation ranged from 700 to 1400 mm (Chen et al., 2011). The mountain vegetation mainly comprises broad‐leaved and mixed forests with a high abundance of Korean pine (*Pinus koraiensis* Siebold & Zucc.) at lower elevation (up to 1100 m) and spruce—fir coniferous forests at higher elevation (up to 1700 m) followed by birch forests and tundra (Tang et al., 2011; Yu et al., 2013). The current study focuses on the forest area between 800 and 1700 m asl, where seven plots of an elevational difference of 150 m were sampled. Every plot was subdivided into four subplots with at least 50 m distance between them (Appendix Figure A1). Samples were taken in early September 2019. Three soil cores of a diameter of 5.5 cm were randomly taken at each subplot, the cores were divided into litter layer, upper (0–5 cm) and lower (5–10 cm) soil layer. The three samples per layer were pooled and considered as one replicate, resulting in four replicates per elevation. Samples were transported in cooling boxes to the laboratory and frozen at −26°C. Prior to further analyses, thawed litter samples were cut into pieces of ca. 2.5 cm × 2.5 cm by scissors, and thawed soil samples were sieved through 2 mm mesh and thoroughly mixed.

### 2.2 Chemical and microbial analyses

Soil and litter pH was measured in 0.01 M CaCl_2_ solution. For carbon and nitrogen analyses 2 g of soil and 1 g of litter were dried at 70°C for 24 h and milled. Aliquots of ca. 1.5 mg of litter and ca. 10 mg of soil were transferred into tin capsules. Carbon and nitrogen content, and natural ^13^C/^12^C isotope ratios (Table 1) were measured using an isotopic mass spectrometer (Delta plus XP, Thermo Electron, Bremen, Germany) coupled via an interface (Conflo III, Thermo Electron, Bremen, Germany) to an elemental analyzer (Flash 2000, Thermo Fisher Scientific, Cambridge, UK). The abundance of ^13^C was expressed as δ values, calculated as $\delta^{13}C\;\left(‰\right)=\frac{R_{\text{sample}}-R_{\text{standard}}}{R_{\text{standard}}}\times 100=\left(\frac{R_{\text{sample}}}{R_{\text{standard}}}-1\right)\times 10^{3}$, with R_sample_ and R_standard_ being the ^13^C/^12^C ratio in the sample and standard. Vienna Pee Dee belemnite was the primary standard for ^13^C. Acetanilide was used as an internal standard.

**TABLE 1:** Mean ± SE of natural δ^13^C [‰] values across elevations and soil layers.

Layer	800 m	950 m	1100 m	1250 m	1400 m	1550 m	1700 m
litter	−27.7 ± 0.38	−28.0 ± 0.39	−27.5 ± 0.36	−28.3 ± 0.77	−27.7 ± 0.43	−27.8 ± 0.61	−27.5 ± 0.60
0–5 cm	−26.5 ± 0.18	−26.6 ± 0.43	−26.4 ± 0.64	−26.5 ± 0.65	−25.9 ± 0.30	−25.7 ± 0.34	−25.8 ± 0.32
5–10 cm	−25.7 ± 0.27	−26.0 ± 0.24	−25.3 ± 0.41	−25.9 ± 0.18	−25.2 ± 0.05	−25.0 ± 0.22	−25.4 ± 0.20

A set of climatic variables retrieved from worldclim2 was ascribed to every elevational plot and extracted via the “raster” package at 30 s resolution (Fick & Hijmans, 2017; Hijmans, 2021). Precipitation and temperature seasonality were calculated as the standard deviation of the yearly precipitation and temperature (mean of monthly means), respectively.

For measuring the concentrations of eleven elements in litter and soil (aluminum, calcium, copper, iron, magnesium, manganese, phosphorus, potassium, sodium, sulfur, zinc), subsamples of the litter layer were dried (60°C, 48 h), milled and digested with 65% HNO_3_ at 195°C for 8 h. For soil layers, the cation exchange capacity was measured from 2.5 g of fresh soil. Samples were saturated with 0.2 N BaCl_2_ overnight, followed by a 4 h percolation phase in which the solved ions were exchanged with Ba^2+^. Extracted ions were analyzed by ICP‐OES (inductively coupled plasma optical emission spectrometry, ICAP 7000 ICP‐OES Analysator, ThermoFisher Scientific).

For measuring microbial respiration and biomass, samples were placed at 4°C for 72 h for thawing prior to the analyses and then preincubated for 7 days at room temperature. A total of 0.8 g of litter and 2 g of each soil layer were used for measuring basal respiration (BR) and substrate‐induced respiration (SIR) following Anderson and Domsch (1978). O_2_ consumption (μl O_2_ g^−1^ soil dw h^−1^) was measured every 0.5 h at 22.0°C using an automated respirometer based on electrolytic O_2_ compensation (Scheu, 1992). For BR, the mean of readings from 6 to 12 h after attachment of the vessels to the respirometer was used. For measuring SIR, a glucose solution was added with 80 mg g^−1^ dry weight added to litter and 8 mg g^−1^ dry weight to soil. The mean of the lowest three measurements of the glucose‐amended samples was used as the maximum initial respiratory response (MIRR; μl O_2_ g^−1^ dry weight h^−1^). Microbial biomass (C_mic_) was calculated as MIRR ⨯ 38 ⨯ 0.7 (Beck et al., 1997). The specific respiration (qO_2_; μl O_2_ mg^−1^ C_mic_ h^−1^) was calculated as a quotient between BR and C_mic_. To facilitate comparisons between soil layers C_mic_ was expressed per gram organic carbon as mg C_mic_ g^−1^ C.

Phospholipids were extracted using a modified high throughput method based on Buyer and Sasser (2012). Lipids were separated through silica columns (0.5 g silicic acid, 3 ml; HF BOND ELUT‐SI, Varian Inc., Darmstadt, Germany). Twenty μl of internal standard (FAME CRM47885, C11 to C24; BAME 47080‐U, C11 to C20; Sigma‐Aldrich, Darmstadt, Germany) was added before the evaporation at the end of lipid extraction. Samples were evaporated for 40 min at 50°C and then at 37°C using a vacuum centrifuge. Then, 0.2 ml transesterification reagent was added and the vials incubated at 37°C for 15 min before adding 0.4 ml of acetic acid (0.075 M) and 0.4 ml chloroform. The lower phase containing the phospholipid methyl esters (FAMEs) was transferred into new vials and the separation step was repeated with another 0.4 ml of chloroform and evaporated at room temperature. As dissolvent for FAMEs, we used 75 μl isooctane. The resulting fatty acid methyl esters were analyzed in a gas‐chromatograph (GC‐FID Clarus 500; PerkinElmer Corporation, Norwalk) equipped with an Elite 5 column (30 m × 0.32 mm inner diameter, film thickness 0.25 μm). The abundance of the lipids was calculated as nmol per gram of dry material and then transformed into mole percentages (Pollierer et al., 2015).

To further characterize the microbial community, the ratio between fungal (18:2ω6,9) and bacterial PLFAs (i15:0, a15:0, i16:0, cy17:0, cy19:0) was calculated (fun/bac ratio) (Moore‐Kucera & Dick, 2008). In addition, the ratio between the sum of cyclopropyl acids (cy17:0, cy19:0) and their monoenoic precursors (16:1ω7, 18:1ω7) was calculated (cyclo/pre ratio) and used as an indicator for physiological stress, e.g., caused by low pH and low nutrient supply (Bossio & Scow, 1997; Guckert et al., 1986; Knivett & Cullen, 1965). Furthermore, we calculated the ratio between saturated (14:0, 15:0, 16:0, 17:0, 18:0) and monounsaturated PLFAs (16:1ω7, 17:1, 18:1ω9, 18:1ω7) (sat/mono ratio), representing nutritional or substrate‐related stress (Bossio & Scow, 1997; Moore‐Kucera & Dick, 2008). Also, the ratio of branched‐chain PLFAs (i15:0, a15:0, i16:0, i17:0), representing Gram^+^ bacteria, and straight monounsaturated PLFAs (16:1ω7, 17:1, 18:1ω9, 18:1ω7), representing Gram^−^ bacteria, were calculated (Gram^+^/Gram^−^ ratio) (Joergensen, 2022; Ratledge & Wilkinson, 1988).

### 2.3 Statistical analyses

Statistical analyses were performed in R v 4.0.4 (R Core Team, 2021). To analyze differences in microbial community composition among elevations and layers, Bray–Curtis distance‐based PERMANOVAs were performed using the “adonis” function. The input matrix included amounts of PLFAs as mole percentages as dependent variables. Elevation, soil layer and their interaction were included as independent factors. Nonmetric multidimensional scaling (NMDS) was used to display differences in PLFA composition in 2‐dimensional space. To identify the PLFAs responsible for most of the variation between elevations and soil layers, the Bray–Curtis distance‐based analysis of similarity percentages (“SIMPER”) was conducted (Oksanen et al., 2020).

To investigate environmental factors structuring the PLFA composition in litter and soil, redundancy analysis (RDA) was used. The response matrix was the same as for the Bray–Curtis distance‐based method described above. While the response matrix was left unscaled, the matrix containing the environmental factors, including local soil factors (including the eleven elements) and climatic factors, was scaled to values between 0 and 1 to secure comparability of effects. RDAs were calculated for all three layers and predictors were selected after correlation and co‐linearity between each other; pH was included in all RDA models since it has been identified as the main structuring force for microbial communities at Changbai Mountain (Shen et al., 2013). With this preselected set of explanatory variables, a permutational, *p*‐value‐based forward selection was run via the “ordistep” function (Oksanen et al., 2020). The number of permutations was 1000. The significance of the variation explained by the selected model and its predictors was tested with the permutational‐based “anova.cca” function and their explanatory impact was analyzed via the adjusted R^2^‐values of the model (Oksanen et al., 2020). The RDA model was displayed as 2‐dimensional biplot of “species”‐scaled values to focus on the impact of the factors characterizing community composition.

Variations in C_mic_ and qO_2_ with elevation and soil layer were inspected using linear mixed‐effects models with plot‐ID as a random term (Bates et al., 2022); if necessary, data were log_10_ transformed to approximate Gaussian distribution. Independent variables were elevation, soil layer and their interaction. If the interaction between elevation and soil layer was significant each layer was analyzed separately, using multiple linear models with the respective dependent variable as mentioned above and elevation as an independent factor. Linear models met the assumption of homoscedasticity and independence. The independent factor elevation was ordered in all analyses. For visualization of pairwise differences in figures, we computed Tukey's honestly significant difference (HSD) using the “emmeans” package (Lenth, 2022). Errors presented in text and figures represent the standard error of the mean (SEM). To gain a better understanding of the observed changes, we correlated factors varying with elevation (C_mic_, qO_2_, cyclo/pre and mono/sat ratios) with the environmental factors, which were identified by forward selection in the RDAs to significantly affect the PLFA patterns (δ^13^C, C/N, pH, Ca concentration, water content) using “Spearman rank correlation” to account for nonlinear relationships revealed by visual inspection.

## 3 RESULTS

### 3.1 Microbial biomass across elevations and layers

**FIGURE 1:**
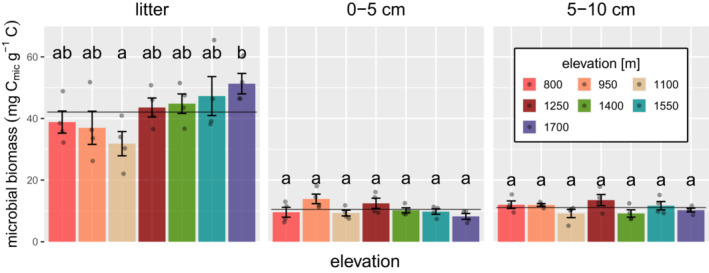
Changes in microbial biomass with elevation in litter, 0–5 and 5–10 cm soil depth. The solid line represents the mean across elevations, the dots represent data points, error bars represent the standard error of the mean and letters mark significant differences between means (Tukey's HSD test at *p* < .05). For results of linear models, see text.

**FIGURE A2:**
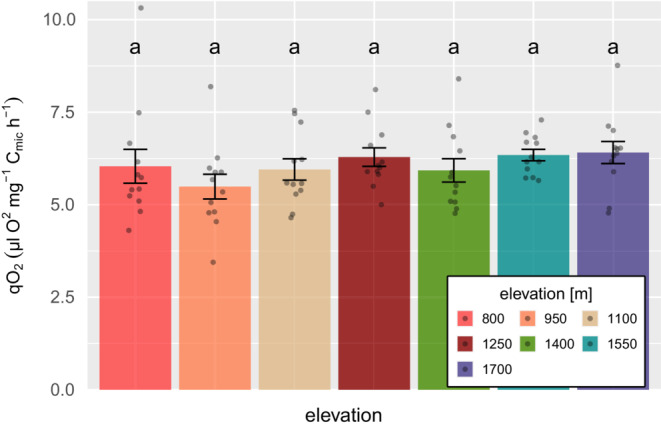
Changes in microbial specific respiration (qO_2_) with elevation. Error bars represent the standard error of the mean, the dots the real data points. Letters mark significant differences between means (Tukey's HSD test at *p* < .05). For results of linear mixed effect model, see text.

To study the expected variations in microbial biomass (C_mic_), we tested the influence of elevation and layer on C_mic_ and their interaction. Microbial biomass varied strongly among layers and generally declined from the litter layer (42.08 ± 1.84 mg C_mic_ g^−1^ C) to 0–5 and 5–10 cm soil by 75% and 73%, respectively, but the decline varied with elevation (significant layer ⨯ elevation interaction; χ^2^ = 24.65, *p* = .017). A separate analysis of each layer showed that C_mic_ only varied significantly with elevation in litter (F_6,21_ = 2.67, *p* = .044), where it first declined from 800 (38.82 ± 3.58 mg C_mic_ g^−1^ C) to 1100 m by 18% and then increased from 1100 m (31.83 ± 3.93 mg C_mic_ g^−1^ C) up to 1700 m by 61% (Figure 1). By contrast, in 0–5 and 5–10 cm soil C_mic_ did not show a clear pattern, but was generally low at 1100 m and high at 1250 m. In contrast to C_mic_, qO_2_ varied significantly with elevation (χ^2^ = 13.95, *p* = .03) but not among soil layers; it was generally low at 950 m (overall mean across layers 5.49 ± 0.33 μl O_2_ mg^−1^ C_mic_ h^−1^) and highest at 1700 m (6.41 ± 0.30 μl O_2_ mg^−1^ C_mic_ h^−1^), but the variations were generally small (Appendix Figure A2).

Correlation analysis between C_mic_ and qO_2_ and environmental factors showed that in litter Ca concentration correlated negatively with C_mic_ and qO_2_ (*ρ* = −.60, *p* < .001 and *ρ* = −.54, *p* = .03, respectively), while C/N ratio correlated positively with C_mic_ and qO_2_ (*ρ* = .38, *p* = .047 and *ρ* = .50, *p* = .006, respectively). Also, pH correlated negatively with C_mic_ in the litter layer (*ρ* = −.61, *p* < .001), reflecting that it was closely inter‐correlated with Ca (*ρ* = .63, *p* < .001). Contrasting the litter, Ca concentrations and pH correlated positively with C_mic_ in 0–5 cm soil (*ρ* = .38, *p* = .045 and *ρ* = .72, *p* < .001, respectively). In 0–5 cm and 5–10 cm, soil water content correlated negatively with C_mic_ (*ρ* = −.39, *p* = .038 and *ρ* = −.42, *p* = .026, respectively).

### 3.2 Microbial community composition across elevations and layers

PLFA profiles as a proxy for microbial community composition varied significantly among layers (F_2,81_ = 98.64, *p* = .001) as well as among elevations (F_6,77_ = 5.05, *p* = .001), with both interacting significantly (F_12,71_ = 2.21, *p* = .006). SIMPER analysis detected the fungal marker 18:2ω6,9 as the most important PLFA separating litter and the two soil layers (Appendix Table A1) decreasing from litter to 0–5 cm by 83% and to 5–10 cm by 85%. The Gram^−^ bacterial marker 18:1ω7 was the PLFA accounting for most of the dissimilarity between 0–5 cm and 5–10 cm, but it decreased from 0–5 cm to 5–10 cm by only 3% reflecting the overall similarity of the PLFA profiles of these two layers.

**FIGURE 2:**
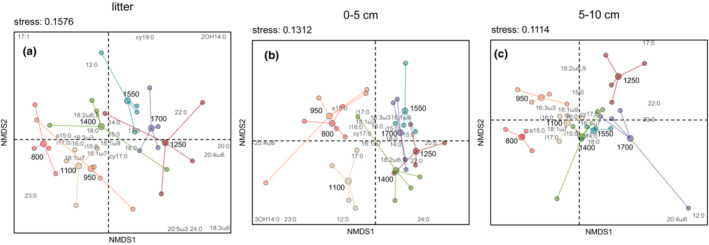
Biplots of two dimensional NMDS of phospholipid fatty acid (PLFA) profiles across elevations (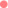 800 m; 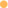 950 m; 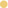 1100 m; 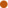 1250 m; 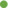 1400 m; 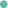 1550 m; 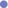 1700 m) in (a) litter, (b) 0–5 cm soil and (c) 5–10 cm soil. Lines connect the elevation centroids to the subplots of the respective elevation.

Due to the significant interaction between layer and elevation, we inspected the layers separately and displayed the PLFA profiles of individual layers (Figure 2). In the litter layer PLFA profiles significantly changed with elevation (F_6,21_ = 2.49, *p* = .007). The sites at 800, 950 and 1100 m separated from those at 1550 and 1700 m, while the PLFA profiles at 1250 and 1400 m largely overlapped with the other elevations (Figure 2a). Although PLFA profiles in 0–5 cm (F_6,21_ = 5.66, *p* < .001) and 5–10 cm (F_6,21_ = 4.81, *p* < .001) also differed significantly with elevation, the differences were less pronounced compared with litter (Figure 2b, c). However, as in litter, the sites at 800, 950, and 1100 m clustered close together, and this also applied to the sites at 1250, 1400, 1550 and 1700 m. In litter, the monounsaturated PLFAs 18:1ω7, 18:1ω9, and 16:1ω7 were more abundant at lower elevations, while the abundance of the saturated fatty acids 14:0, 16:0, and 17:0 peaked at 1550 and 1700 m (Figure 2a). PLFA 16:0 accounted for most of the dissimilarity between 800 and 1700 m (4.35%), and PLFA 18:1ω7 accounted for the second most dissimilarity (3.08%; Appendix Table A2). In 0–5 cm depth, the PLFA pattern generally resembled that in litter, however, the PLFA accounting for most of the dissimilarity between 800 and 1700 m was 18:1ω7 (2.41%; Appendix Table A2). In 5–10 cm depth, 800 m separated from the higher elevations, being most dissimilar to 1700 m (group dissimilarity 10.56%), with PLFA a15:0 accounting for most of the dissimilarity (2.34%, Appendix Table A2). Branched‐chain PLFAs a15:0, i16:0 and cy17:0 were associated with 800, 950 and 1100 m, while the unsaturated PLFAs 18:2ω6,9 and 18:1ω7 were most abundant at 1400, 1550 and 1700 m.

**FIGURE 3:**
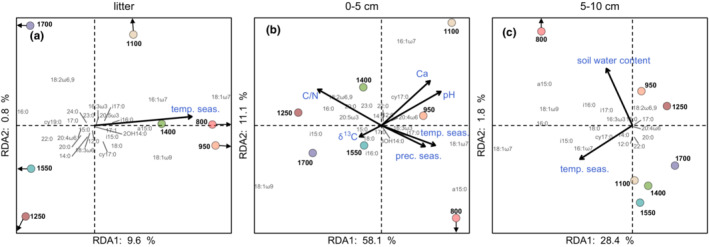
RDA biplots on the relationship between phospholipid fatty acids and significant environmental factors (pH, temperature seasonality, precipitation seasonality, δ^13^C of the respective layer and soil water content of the respective layer; identified by P‐value based forward selection) in (a) litter, (b) 0–5 and (c) 5–10 cm soil depth. Centroids of elevations are represented by colored dots (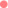 800 m; 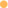 950 m; 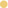 1100 m; 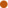 1250 m; 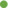 1400 m; 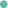 1550 m; 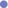 1700 m). The variation explained by the RDA axes is given as percentages of total. Elevations marked with arrows are positioned beyond the plotted borders of the RDA axes; arrows point in the direction of their position. All coordinates were species‐scaled.

As indicated by RDA, the environmental factors that correlated with certain PLFAs varied between layers (Table 2, Figure 3) and this was also true for the variance in PLFA patterns explained by RDA axes 1 and 2 in litter, 0–5 and 5–10 cm depth (10.4%, 69.2% and 30.2%, respectively). Temperature seasonality was the only significant environmental variable in the litter layer and was highest at 800 and 950 m. In the litter layer, the monounsaturated PLFAs 16:1ω7, 18:1ω7 and 18:1ω9 increased with temperature seasonality (Figure 3a). In 0–5 cm depth, C/N ratio, pH, Ca concentrations, δ^13^C, precipitation seasonality and temperature seasonality significantly explained the PLFA distribution (Table 2, Figure 3b). PLFA 16:0 increased with the C/N ratio and reached a maximum at intermediate elevations (1250 and 1400 m). Soil pH and Ca concentrations increased towards lower elevations parallel to PLFA cy17:0. Temperature seasonality and precipitation seasonality increased parallel to PLFA a15:0 reaching a maximum at 800 m. δ^13^C reached a maximum of 1550 and 1700 m. In 5–10 cm depth, soil water content and temperature seasonality significantly explained the variation in the PLFA distribution (Table 2, Figure 3c). Temperature seasonality increased with the PLFAs cy17:0 and i15:0, and the soil water content increased with the Gram^+^ marker PLFAs i16:0 and i17:0, and PLFA a15:0 being highest at 1250, 950 and 800 m, respectively.

**TABLE 2:** F‐ and p‐values for pH, Ca concentrations, C/N ratio, δ^13^C values, water content, temperature seasonality and precipitation seasonality as predictors of PLFA patterns in litter, 0–5 and 5–10 cm soil as analyzed by RDA and presented in Figure 3.

Predictor	Litter layer		0–5 cm		5–10 cm	
	F‐value	*p*‐value	F‐value	*p*‐value	F‐value	*p*‐value
pH	0.45	.66	24.03	<.001	0.64	.468
Ca	—	—	3.56	.027	—	—
C/N	—	—	50.38	<.001	—	—
δ^13^C	—	—	6.44	.003	—	—
Water content	—	—	—	—	8.21	.007
Temperature seasonality	4.71	.008	4.87	.014	5.44	.019
Precipitation seasonality	—	—	5.00	.010	—	

*Note*: Environmental factors were chosen via *p*‐value based forward selection per layer. All selected factors are displayed; “—” indicates that factors were not chosen for the respective layer. pH was included in all RDAs due to its importance for microbial community composition shown in a previous study at Changbai Mountain (Shen et al., 2013).

### 3.3 Indicators of community changes and nutritional stress

**FIGURE 4:**
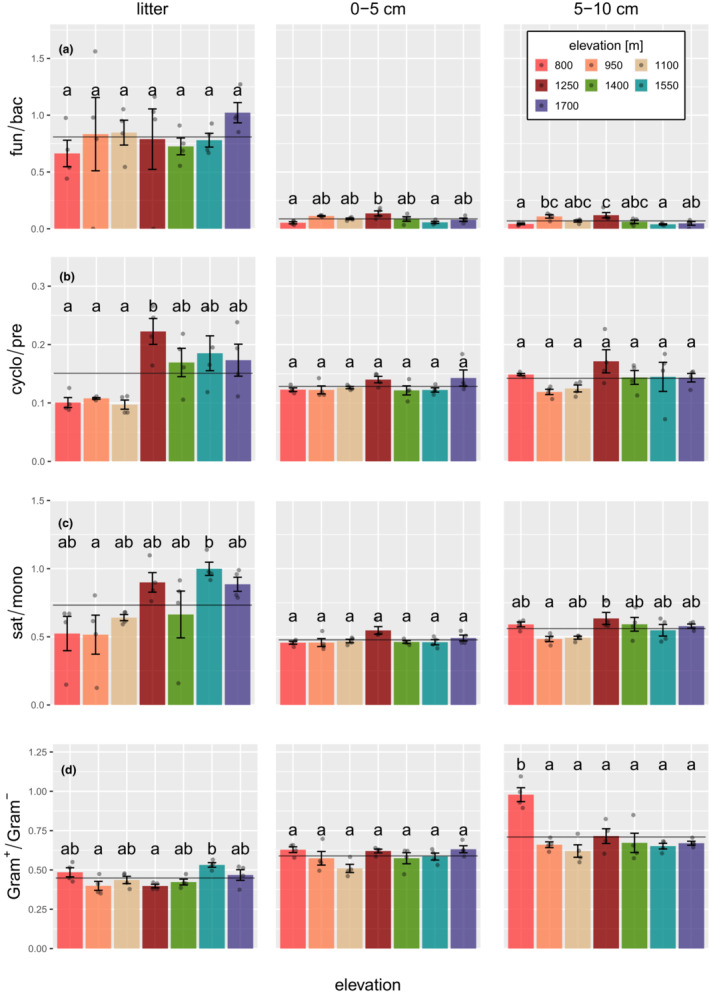
Changes in the (a) fungal/bacterial (fun/bac), (b) cyclopropyl/monoenoic (cyclo/pre), (c) saturated/monounsaturated (sat/mono) and (d) Gram^+^/Gram^−^ marker PLFA ratios (Gram^+^/Gram^−^) in litter, 0–5 and 5–10 cm soil depth with elevation. The solid line represents the overall mean, the dots the data points. Error bars represent the standard error of the mean and letters mark significant differences between means (Tukey's HSD test at *p* < .05). For results of linear models see text.

Of the four common PLFA indicator ratios, the fun/bac PLFA ratio significantly decreased from litter (overall mean 0.8 ± 0.18) to 0–5 and 5–10 cm depth by 89.2% and 91.4%, respectively (χ^2^ = 278.58, *p* < .001; Figure 4a); it did not vary significantly with elevation. The cyclo/pre ratio, as a measure of physiological stress, significantly decreased from litter (overall mean 0.15 ± 0.01) to 0–5 and 5–10 cm by 14.9% and 5.7%, respectively (χ^2^ = 10.25, *p* = .006; Figure 4b); however, the decline depended on elevation (significant elevation ⨯ layer interaction; χ^2^ = 40.96, *p* < .001). As indicated by separately analyzing the three layers, the cyclo/pre ratio only varied significantly with elevation in litter (F_6,21_ = 5.86, *p* = .001), where it was high at elevations ≥1250 m and low at elevations ≤1100 m. Variations in the sat/mono ratio, a measure for substrate‐induced stress, also depended on both soil layer and elevation (significant elevation ⨯ layer interaction; χ^2^ = 25.92, *p* = .011), and followed a very similar pattern to the cyclo/pre ratio (Figure 4c). In contrast to the cyclo/pre ratio, however, the separate analysis of the three layers indicated that the sat/mono ratio changed significantly with elevation in each of the layers being high at elevations ≥1250 m and low at elevations ≤1100 m in litter (F_6,21_ = 3.45, *p* = .016), and at a maximum at 1250 m in 0–5 (F_6,21_ = 2.67, *p* = .044) and 5–10 cm depth (F_6,21_ = 2.93, *p* = .031). In contrast to the other three ratios, the Gram^+^/Gram^−^ ratio generally increased from litter to 0–5 and 5–10 cm depth by 23.7% and 63.4%, respectively (χ^2^ = 73.83, *p* < .001; Figure 4d); however, again the effect of layer depended on elevation (significant elevation ⨯ layer interaction; χ^2^ = 25.92, *p* = .011). As indicated by separately analyzing the three layers, changes with elevation were only significant in litter (F_6,21_ = 4.23, *p* = .006) and 5–10 cm depth (F_6,21_ = 10.09, *p* < .001), but in trend also in 0–5 cm depth (F_6,21_ = 2.47, *p* < .058). In litter, the Gram^+^/Gram^−^ ratio was highest at 1550 m and lowest at 950 and 1250 m, whereas in 0–5 cm depth it was similarly high at 800, 1250 and 1700 m, and lowest at 1100 m, and in 5–10 cm depth it was highest at 800 m and again lowest at 1100 m.

Subsequent correlation analysis indicated that in litter Ca concentrations correlated strongly negatively with the sat/mono and cyclo/pre ratios (*ρ* = −.70, *p* < .001 and *ρ* = −.72, *p* < .001, respectively). As being closely inter‐correlated, pH correlated positively with the sat/mono and cyclo/pre ratios in litter (*ρ* = .49, *p* = .008 and *ρ* = −.56, *p* = .002, respectively). Further, the C/N ratio correlated positively with the cyclo/pre ratio in litter (*ρ* = .49, *p* = .008), but negatively with the Gram^+^/Gram^−^ ratio (*ρ* = −.56, *p* = .002). As in litter, Ca concentrations in 0–5 cm soil depth correlated negatively with the sat/mono and cyclo/pre ratios (*ρ* = −.50, *p* = .007 and *ρ* = −.54, *p* = .003, respectively), and for the sat/mono ratio this was also true for pH (*ρ* = −.44, *p* = .021). Further, the C/N ratio correlated positively with the sat/mono and cyclo/pre ratios (*ρ* = .68, *p* = < .001 and *ρ* = .45, *p* = .015, respectively), while pH correlated negatively with the Gram^+^/Gram^−^ ratio (*ρ* = −.57, *p* = .002). In 5–10 cm soil depth, only Ca content correlated negatively with the cyclo/pre ratio (*ρ* = −.39, *p* = .042).

## 4 DISCUSSION

Using a combination of respiration‐based parameters and PLFA patterns, we identified variations in microbial community composition and functioning in litter and soil of natural forests across an elevational gradient at Changbai Mountain. The results showed strong variations in microbial communities between litter and soil along the studied elevational gradient. Further, the results indicate that the factors responsible for the changes in the structure and functioning of microbial communities also differed between layers. Only temperature seasonality affected the PLFA patterns in a uniform way across soil layers.

Supporting our first hypothesis, C_mic_ strongly decreased from litter to 0–5 and 5–10 cm soil depth, presumably reflecting the decrease in resource availability from litter to deeper soil layers. However, qO_2_ did not differ significantly between soil layers suggesting that the efficiency in the use of carbon resources by microorganisms is similar across soil layers (Cao et al., 2019). Interestingly, C_mic_ responded differently to the elevational gradient in litter and soil. In litter, C_mic_ increased with increasing elevation above 1100 m and correlated negatively with the concentration of Ca along the elevational gradient. Ca is involved in a number of bacterial processes, one of the most important being the recovery of nitrogen from urea via urease reaction (Castanier et al., 1999; Krajewska, 2018). The role of Ca and the contribution of microorganisms to the cycling of nitrogen has been investigated in detail in arable soils (Bowles et al., 2014; Klose & Tabatabai, 2000), while its role in forest soils remains little studied. Klose and Tabatabai (1999) found urease activity to be mainly of microbial origin in a variety of soils, underlining the potential influence of Ca on the mobilization of nitrogen and microbial nitrogen nutrition.

The increases in C_mic_ and in part of qO_2_ towards higher elevations, and its (strongly) negative correlation with Ca (and pH) and (moderately) positive correlation with litter C/N ratio may reflect that nutritional shortage is more pronounced in communities of high C_mic_ and microbial activity. In fact, microbial activity can increase with stronger nitrogen limitation and decrease with the addition of nitrogen (Averill & Waring, 2018; Craine et al., 2007) following the “microbial nitrogen mining” hypothesis (Moorhead & Sinsabaugh, 2006). Wild et al. (2017) showed that a short‐term input of carbon increases microbial growth and the microbial demand for nitrogen, but does not influence nitrogen mining. Therefore, high carbon availability at high elevations may also explain the positive correlation between PLFA stress indicators and litter C/N ratio due to increased nitrogen demand and increased C_mic_. In addition, high Ca concentrations at lower elevations may facilitate microbial nitrogen acquisition and therefore result in lower microbial stress. Overall, our first hypothesis was only supported in part; in the litter layer, C_mic_ responded as hypothesized, even though not linear, with the main drivers being variations in the availability of carbon and nitrogen but also Ca along the elevational gradient, while C_mic_ in the two soil layers was rather constant across elevations.

Supporting our second hypothesis, PLFA profiles clearly separated the three layers, and this was mainly due to the decrease in fungal PLFA markers from the litter to the two soil layers and the decrease in Gram^−^ bacterial markers from 0–5 to 5–10 cm. This is in line with the results of the study by Šnajdr et al. (2008), who documented a rapid decrease in fungal biomass from the litter to the fermentation layer in forests. Fungi are known to be the major decomposers of recalcitrant carbon compounds and typically dominate in the litter layer, while bacteria play a larger role in the decomposition of root exudates thereby dominating in soil (de Boer et al., 2005). Further, the Gram^+^/Gram^−^ ratio increased with soil depth, since Gram^−^ bacteria heavily depend on plant‐derived carbon, such as litter, while Gram^+^ bacteria preferentially use soil organic matter‐derived carbon (Kramer & Gleixner, 2008).

PLFA profiles in the litter layer also varied with elevation, and temperature seasonality was the only environmental variable studied significantly affecting them, which is in line with our third hypothesis, even though we expected more climatic variables to influence PLFA profiles in litter. Temperature seasonality represents the variation in temperature during the year and litter is more heavily exposed to such fluctuations in temperature than deeper soil layers.

Generally, increasing temperature accelerates the decomposition of litter (Kirschbaum, 1995) resulting in more shallow organic layers (Raich et al., 2006). Associated with higher temperatures, the decomposition rates of forest litter typically increase towards lower elevations (Salinas et al., 2011). However, in addition to the increase in temperature at lower elevations, temperature variation within the year also increases at lower elevations, and the vegetative period starts earlier and lasts longer compared with higher elevations. In spring decomposition rates of litter strongly increase (Kreyling et al., 2013), but at higher elevations this is less pronounced resulting in litter accumulation and reduced nutrient mobilization. At Changbai Mountain, coniferous stands of spruce and fir dominate at elevations between 1100 and 1700 m (Tang et al., 2011; Yu et al., 2013). Coniferous needles contain high amounts of lignin and polyphenols (Gallet & Lebreton, 1995; Taylor et al., 1989), thereby typically decomposing more slowly than deciduous litter (Prescott, 2010).

Although litter decomposition is hampered during winter it does not stop and may benefit from snow cover preventing or reducing the freezing of litter and soil (Kreyling et al., 2013; Schimel et al., 2004, 2007). Uchida et al. (2005) reported that 26% of the annual mass loss of litter occurs under snow. Notably, the interception of snow by trees is higher, and therefore, snow cover is sparser in evergreen coniferous compared with deciduous forests (Noguchi & Nishizono, 2010; Vikhamar & Solberg, 2003). This reduced snow cover, which is related to low‐temperature seasonality, together with low‐quality needle litter may explain the accumulation of litter at high elevations, while the opposite may be true at low elevations, with these differences likely affecting microbial biomass and community structure. Neither pH nor Ca significantly explained variations in PLFA profiles in litter, but the negative correlations of PLFA stress ratios with litter pH and concentrations of Ca indicate that microbial communities and their functioning is in fact affected by the availability of base cations.

Changes in microbial parameters in 0–5 and 5–10 cm soil depth were similar and differed strongly from those in litter. Of the studied environmental factors, only temperature seasonality structured microbial community composition across layers. In 0–5 cm depth the C/N ratio of the soil explained a large fraction of the variations in PLFA profiles supporting our third hypothesis. This is in line with the findings of Liu et al. (2019) that carbon and nitrogen concentrations strongly affect PLFA patterns at Changbai Mountain, however, compared with the current study, they only investigated soil layers of a smaller elevational gradient comprising only pine forests. Notably, the C/N ratio in 0–5 cm depth was much lower than in the litter layer indicating increased microbial access to nitrogen. Across the elevational gradient, the C/N ratio in 0–5 cm depth was highest at 1250 m and this was associated with an increase in PLFA 15:0 and an increased sat/mono ratio pointing to nutritional stress at this nitrogen‐poor site. Additionally, C_mic_ was high at 1250 m and, as in the litter layer, this may have aggravated nitrogen limitation (Dubinkina et al., 2019). Another soil‐related factor that correlated with microbial community structure in 0–5 cm depth was δ^13^C values of soil organic matter. δ^13^C increased towards higher elevations indicating an increasing state of decomposition of organic matter (Melillo et al., 1989; Potapov et al., 2019), related to high microbial biomass and activity in litter. Other soil factors driving the PLFA composition in 0–5 cm depth were Ca concentrations and pH, which increased towards lower elevations, indicating again that the effect of pH on microbial community composition at our study sites is not linked to physiological stress by acidity but the abundance of base cations. Variations in Ca concentrations rather than pH itself may be responsible for the widely reported correlation between pH and the structure of microbial communities (Högberg et al., 2007; Männistö et al., 2007; Zhou et al., 2017).

Besides these local soil‐related factors, temperature seasonality explained a large fraction of the variation in PLFA profiles in 0–5 and 5–10 cm depth, and in 0–5 cm also precipitation seasonality, contrasting the litter layer. Changbai Mountain has a rather constant warm climate during the relatively short vegetative period followed by harsh winters with mean monthly temperatures below −20°C in January (Yu et al., 2013). Temperature and precipitation seasonality increase towards lower elevations, reflecting longer and warmer summers as well as more pronounced seasonality at lower elevations. In particular marker PLFAs for Gram^+^ bacteria increased towards lower elevations, especially in 5–10 cm depth, where the Gram^+^/Gram^−^ ratio was highest at 800 m. Due to their strong and interlinked peptidoglycan cell walls, Gram^+^ bacteria are more resistant to temperature and moisture changes than Gram^−^ bacteria (Schimel et al., 2007). Interestingly, the Gram^+^/Gram^−^ decreased from 800 to 1100 m and this was most pronounced in 5–10 cm depth. Gram^−^ bacteria depend more heavily on labile carbon resources, while Gram^+^ bacteria can access more recalcitrant carbon compounds (Fanin et al., 2019; Kramer & Gleixner, 2008). High microbial activity and biomass due to high temperatures during the vegetative period may hamper the leaching of labile carbon compounds into the soil, which is supported by the strong increase in the Gram^+^/Gram^−^ ratio from litter to 0–5 and 5–10 cm soil depth. Notably, we took our samples in September before the deciduous trees shed their leaves, and the litter layer comprised predominantly leaf litter material of the previous year depleted in labile compounds, which may have contributed to the low availability of labile carbon compounds in soil and therefore to the increase in Gram^+^ bacteria in soil at 800 m.

The identified effects of temperature seasonality on the structure and functioning of microbial community in each of the layers are of special relevance for the response of decomposer systems and decomposition processes to global warming, which is expected to be associated with increased seasonal temperature fluctuations (Tian et al., 2015). Transplantation experiments along elevational gradients showed decomposition to increase in litter translocated to lower elevations (Salinas et al., 2011), where temperature and temperature seasonality are higher. Therefore, climate change may affect in particular the functioning of microbial communities at high elevations with potential detrimental consequences for carbon sequestration.

Contrasting our fourth hypothesis both stress indicator ratios were highest in the litter layer, but their response also depended on elevation. The moderate positive correlation of the litter C/N ratio with the cyclo/pre ratio in the litter indicates an increase in nutritional stress under high C/N ratios, especially at 1250 and 1400 m. In litter, temperature seasonality correlated positively with PLFAs 18:1ω7 and 16:1ω7 indicating lower microbial stress at lower elevations with higher temperature seasonality, which is further supported by the increase in the sat/mono and cyclo/pre ratio. Besides being an indicator of nutritional stress, the cyclo/pre ratio increased in *E. coli* with the acidity of the environment (Knivett & Cullen, 1965; Moore‐Kucera & Dick, 2008). Conform to these findings, at our study sites the cyclo/pre ratio in the litter layer was negatively correlated with pH and Ca concentrations. Both pH and Ca concentrations changed with the transition from pine to spruce forests at 1100 m, potentially explaining the changes in stress indicator ratios likely due to reduced Ca concentrations and increased litter C/N ratio.

## 5 CONCLUSION

Our study aimed at uncovering variations in microbial community composition and functioning along a natural elevational gradient of forests and identifying the factors responsible for these variations. We identified temperature and precipitation seasonality as major climatic factors driving microbial communities in litter and soil, which is likely due to the pronounced difference between harsh winters, and constant warm and wet summers at Changbai Mountain. Besides climatic factors, the availability of resources played a critical role in structuring microbial communities in litter and upper soil, as indicated by δ^13^C values reflecting the stage of organic matter decomposition and C/N ratio reflecting the availability of nitrogen. The effect of Ca concentrations and pH on the microbial community in upper soil might be linked to nitrogen acquisition via urease reaction. This, however, needs to be proven in future studies measuring both urease activity and Ca concentrations. Pronounced changes in microbial biomass and stress indicator ratios in the litter layer between 1100 and 1250 m indicate a prominent shift in the structure and functioning of microbial communities between pine‐dominated and spruce‐dominated forests. Montane forests are increasingly threatened due to global warming and increased infections by herbivore pest species, and therefore, there is a need to better understand their functioning and regulatory forces including feedback between the below‐ and aboveground system. The present study forms a starting point for such studies. Future studies need to include other soil food web components including soil invertebrates and their relationship with tree species and forest types.

## AUTHOR CONTRIBUTIONS

**Johannes Lux:** Conceptualization (supporting); data curation (lead); formal analysis (lead); investigation (lead); visualization (lead); writing – original draft (lead). **Zhijing Xie:** Writing – review and editing (equal). **Xin Sun:** Conceptualization (supporting); writing – review and editing (equal). **Donghui Wu:** Conceptualization (lead); funding acquisition (lead); project administration (lead); resources (equal); writing – review and editing (equal). **Stefan Scheu:** Conceptualization (lead); funding acquisition (lead); project administration (lead); resources (equal); supervision (lead); writing – review and editing (lead).

## FUNDING INFORMATION

Open access funding enabled and organized by project DEAL.

## CONFLICT OF INTEREST

The authors declare no conflict of interest.

## Data Availability

PLFA mole percentages, environmental factors, microbial respiration and biomass data are accessible via Dryad; DOI: https://doi.org/10.5061/dryad.zs7h44jct.

## APPENDIX A

**TABLE A1:** Pairwise group dissimilarities of PLFA profiles between soil layers.

Contrast	PLFA	Group dissimilarity [%]	Dissimilarity [%]	Mean a [mole percent]	Mean b [mole percent]	Cumsum [%]
0–5 vs 5–10	18:1ω7	9.55	1.61	23.68	22.90	16.89
	18:1ω9		1.43	12.95	10.47	31.84
	a15:0		1.13	6.59	8.41	43.69
	16:0		0.66	15.53	16.32	50.55
	16:1ω7		0.62	9.03	8.28	57.03
	i15:0		0.62	9.79	10.67	63.52
	18:2ω6,9		0.49	2.03	1.82	68.68
	18:00		0.44	3.65	4.35	73.30
0–5 vs litter	18:2ω6,9	24.52	5.10	2.03	11.94	20.79
	18:1ω7		4.25	23.68	15.20	38.13
	16:00		4.23	15.53	20.66	55.39
	i15:0		1.95	9.79	5.91	63.32
	18:1ω9		1.57	12.95	15.16	69.71
	a15:0		1.52	6.59	3.55	75.92
5–10 vs litter	18:2ω6,9	26.83	5.19	1.82	11.94	19.35
	16:00		3.93	16.32	20.66	33.98
	18:1ω7		3.88	22.90	15.20	48.45
	a15:0		2.43	8.41	3.55	57.49
	i15:0		2.38	10.67	5.91	66.36
	18:1ω9		2.35	10.47	15.16	75.13

(Dis‐) similarity percentages (SIMPER analysis) between groups representing the contribution of each PLFA to total dissimilarity (as percentages). The averages of PLFAs of contrasts are given in mole percent. The cumsum displays the additive contribution of PLFAs to the group dissimilarity as percentage of group dissimilarity (up to 70%).

**TABLE A2:** Pairwise group dissimilarities of PLFA profiles between 800 and 1700 m in litter, 0–5 and 5–10 cm soil depth.

Layer	PLFA	Group dissimilarity [%]	Dissimilarity [%]	Mean 800 m [mole percent]	Mean 1700 m [mole percent]	Cumsum [%]
litter	16:0	19.41	4.35	14.87	23.57	22.43
	18:1ω7		3.08	18.83	12.68	38.28
	18:2ω6,9		2.09	11.32	13.98	49.08
	18:1ω9		1.48	15.49	13.77	56.71
	16:1ω7		1.33	10.63	7.97	63.56
	22:0		1.15	0.43	2.72	69.47
	a15:0		1.03	5.11	3.06	74.76
0–5 cm	18:1ω7	10.28	2.41	26.29	21.48	23.41
	a15:0		1.83	9.34	5.68	41.21
	18:1ω9		1.52	12.08	15.11	55.96
	i15:0		0.98	8.66	10.62	65.48
	16:1ω7		0.59	7.48	8.66	71.19
5–10 cm	a15:0	10.82	2.29	11.99	7.40	21.21
	18:1ω7		1.40	19.89	22.68	34.10
	18:1ω9		1.00	11.63	9.64	43.32
	12:0		0.87	0.00	1.73	51.32
	i16:0		0.72	4.47	3.03	57.98
	22:0		0.72	0.24	1.67	64.59
	16:1ω7		0.59	7.23	8.37	70.08

(Dis‐) similarity percentages (SIMPER analysis) between groups as percentages. Dissimilarity represents the contribution in dissimilarity of each PLFA. The averages of PLFAs of 800 m and 1700 m are given in mole percent. The cumsum displays the additive contribution of PLFAs to the group dissimilarity as percentage of group dissimilarity (up to 70%).
