# Correction to Changes in microbial community structure and functioning with elevation are linked to local soil characteristics as well as climatic variables

**DOI:** 10.1002/ece3.11359

**Published:** 2024-05-20

Lux, J., Xie, Z., Sun, X., Wu, D., & Scheu, S. (2022). Changes in microbial community structure and functioning with elevation are linked to local soil characteristics as well as climatic variables. *Ecology & Evolution*, e9632. https://doi.org/10.1002/ece3.9632

We discovered a minor mistake in the Materials and Methods section of our published *Ecology & Evolution* Article, we stated the wrong marker lipids for Gram‐negative bacteria (but used the correct ones in all analyses). Instead of the (wrongly) stated PLFAs 16:1ω7, 17:1, 18:1ω9, and 18:1ω7, we used and analyzed (the correct) cy17:0, cy19:0, 16:1ω7, and 18:1ω7 as markers for Gram^−^ bacteria. Even though this error can be easily identified as copying mistake, we think it might be justified to place a corrigendum due to wrong implications readers may draw from this sentence.

The sentence in this paper reads:

“Also, the ratio of branched‐chain PLFAs (i15:0, a15:0, i16:0, i17:0), representing Gram^+^ bacteria, and straight mono‐unsaturated PLFAs (16:1ω7, 17:1, 18:1ω9, 18:1ω7), representing Gram^−^ bacteria, were calculated (Ratledge & Wilkinson, 1988; Joergensen, 2022).”

It should be replaced by:

“Also, the ratio of branched‐chain PLFAs (i15:0, a15:0, i16:0, i17:0), representing Gram^+^ bacteria (except Actinobacteria), and cyclopropyl and vaccenic type PLFAs (cy17:0, cy19:0, 16:1ω7, 18:1ω7), representing Gram^−^ bacteria, were calculated (Joergensen, 2022; Ratledge & Wilkinson, 1988).”

We apologize for this error.

Sincerely

Johannes Lux (on behalf of all authors)

